# Gamification in Therapeutic Rehabilitation of Distal Radial and Ulnar Fracture: A Case Report

**DOI:** 10.7759/cureus.28586

**Published:** 2022-08-30

**Authors:** Waqar M Naqvi, Moh'd Irshad Qureshi

**Affiliations:** 1 Physiotherapy, Ravi Nair Physiotherapy College, Datta Meghe Institute of Medical Sciences, Wardha, IND; 2 Research, N.K.P. Salve Institute of Medical Sciences and Research Center, Nagpur, IND; 3 Neuro-Physiotherapy, Ravi Nair Physiotherapy College, Datta Meghe Institute of Medical Sciences, Wardha, IND

**Keywords:** oculus quest, hand functions, virtual reality, case report, physiotherapy intervention, functional independence, distal radial and ulnar fracture, gamification

## Abstract

Gamification is a novel interventional approach to functional recovery and rehabilitation. We present a novel gamification rehabilitation case of a 38-year-old female referred to the physiotherapy department for post-distal radial fracture rehabilitation. She was managed with closed reduction and internal fixation (CRIF) using two Kirschner wires (K-wires) after falling on an outstretched hand in a road traffic accident with a two-wheeler. Gamification intervention was provided to the patient for four weeks. We used pre- and post-intervention outcome measures for pain, range of motion, grip strength, and hand function. There was a substantial improvement in all outcome measures after four weeks of gamification intervention.

## Introduction

Distal radius fractures (DRF) are one of the most frequent fractures worldwide and the most common encounter for rehabilitation in physiotherapy practice [1]. Conventional physiotherapy, including cryotherapy, exercise balls, manual therapy, and electrotherapy, remains a common practice in the physiotherapy profession [2]. With the recent upgradation in modern science, technology, and healthcare delving into each other, virtual and augmented reality have become integrated parts of rehabilitation programs for musculoskeletal and neurological conditions [3]. Gamification is a novel interventional approach to functional recovery and rehabilitation by employing elements of games in the process of rehabilitation by using a goal-oriented, 3-D immersive gaming interface approach. It has a significant impact on non-traumatic conditions and chronic neurological and musculoskeletal illnesses; however, the implication of gamification on the functional recovery of patients with DRF is yet to be explored [4]. Although there are various serious gaming or gamification approaches that have recently been used in a variety of musculoskeletal disorders for rehabilitation, gamification using Oculus Quest (Facebook Technologies, Menlo Park, USA) is the most recent and advanced head-mounted display (HMD) for creating a virtual environment for gaming enthusiastic and therapists worldwide are exploring the best possible customized gaming on it for patient rehabilitation programs [5].

## Case presentation

**Figure 1:**
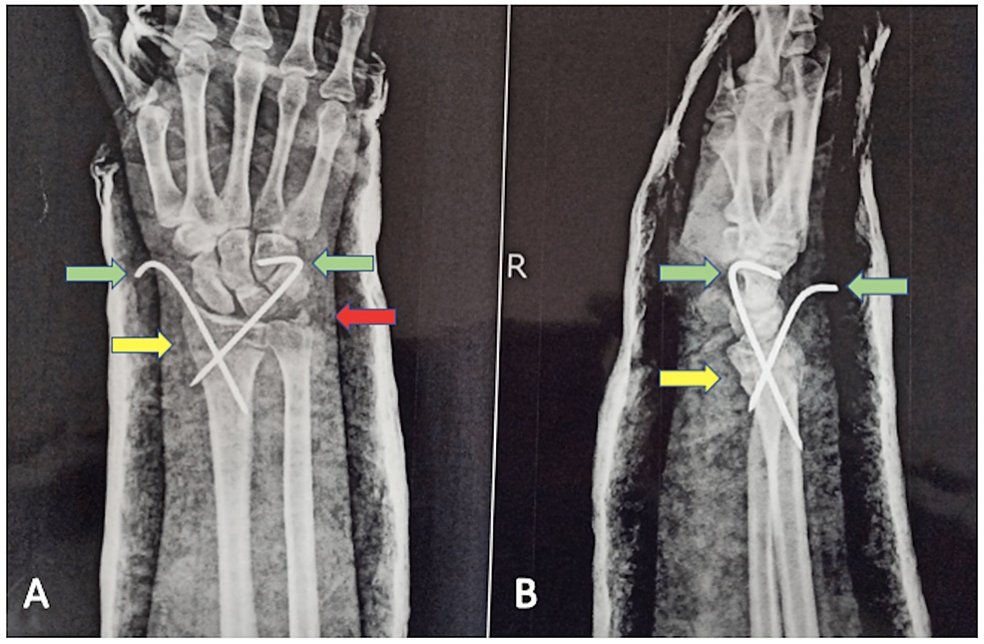
The yellow arrow indicates the line of distal radius fracture in A and B; the red arrow is showing the ulnar styloid fracture in A; and the green arrow is pointing at the K-wires used for fixation in closed reduction internal fixation of the distal radius and ulnar fracture in A and B.

A 38-year-old female approached the physiotherapy outpatient department (OPD) for rehabilitation after a distal radius and ulnar fracture which was managed with closed reduction and internal fixation (CRIF) using two Kirschner wires (K-wires) as shown in Figure 1, after falling on an outstretched hand in a road traffic accident with a two-wheeler. She visited the orthopedic OPD with a complaint of pain in the right wrist, leading to a restricted range of motion (ROM) for an hour. On the initial radiographic investigation, there was a type II distal radial fracture with an ulnar styloid, according to the Frykman classification for distal radial fractures. Subsequently, the patient underwent CRIF with two K-wires (one from the lateral aspect and the other from the dorsal aspect) for the surgical management of the fracture under the axillary nerve block. K-wires were used to maintain traction and alignment angulation of the distal radius; however, the ulnar styloid was conservatively managed. The patient’s hand was placed under a plaster cast extending from the thenar eminence to immediately below the elbow. The hand was immobilized in a cast with a sling pouch for six weeks. The K-wire and cast were removed and physiotherapy rehabilitation was prescribed.

The patient was referred to the physiotherapy OPD on the 40th day after the fall with complaints of pain and restricted ROM in the right wrist following CRIF with K-wires. The pain assessed on patient presentation was continuous and 3.2 on the visual analog scale (VAS) during rest and 7.1 during activity with grade 2 tenderness over the radial and ulnar styloid upon palpation. Upon observation, the swelling was present and the hand was placed close to the body with the elbow flexed in a guarded sling pouch. A fresh wound was present after K-wire removal along with tropical changes. 

On the affected side, the wrist ROM was restricted as shown in the baseline reported readings in Table 1. The grip strength was recorded using a Jamar handheld dynamometer which was 4 on the first day of assessment. We also recorded functional independence of the hand and upper limb using the Disabilities of the Arm, Shoulder, and Hand questionnaire (DASH), a valid and reliable patient-reported outcome measure (PROM) [6], which was 76.5. 

**Table 1:** Improvement in the range of motion (ROM) recorded at the baseline, end of 2nd week, and end of the 4th week with the use of gamification in therapeutic rehabilitation.

Wrist Movement	Baseline	At the end of 2nd week	At the end of the 4th week
Flexion	0-20°	0-40°	0-65°
Extension	0-10°	0-35°	0-60°
Ulnar deviation	0-14°	0-14°	0-48°
Radial deviation	Complete restriction	0-10°	0-18°
Pronation	0-20°	0-30°	0-65°
Supination	0-35°	0-48°	0-60°

**Figure 2:**
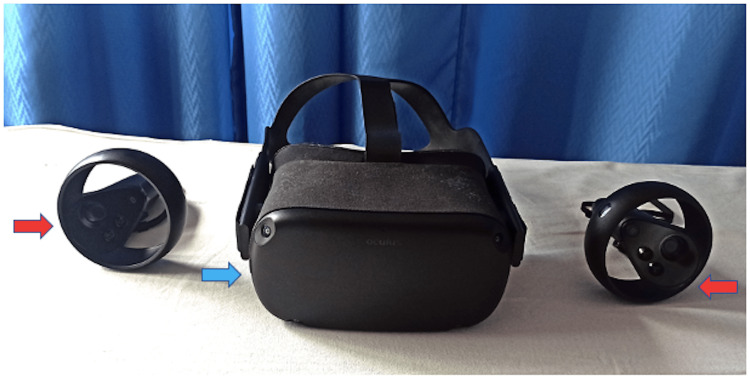
Oculus Quest used for inducing gamification with head-mounted display highlighted using blue arrow with handheld sensors demonstrated using red arrows Oculus Quest, Facebook Technologies, Menlo Park, USA.

The patient underwent a physiotherapy program initially with cryotherapy for 10 minutes to reduce swelling and free hand grip exercise using the exercise ball. The patient was introduced to Oculus Quest, which is a head-mounted display (as shown in Figure 2 with a blue arrow) that creates a virtual environment for gaming, and taught to move the forearm, wrist, and fingers using handheld sensors (as shown in Figure 2 with red arrows) actively from the position at rest to maximally attaining the movements across all axes and planes namely pronation and supination of the forearm, flexion/extension and radial/ulnar deviation of wrist and flexion/extension, thumb abduction/adduction of fingers. Rehabilitation games aimed to help patients perform the above-mentioned exercises through the games developed solely for the rehabilitation of patients following DRF. The games used were 3D immersive representations of contour, position, and movement combined with gestural control in the sensor motion virtual environment. These games provide an interactive, engaging, interesting, and real-time enhancement of movement using a cognitive training technique during rehabilitation. The games included were *Racket: NX *game (One Hamsa, Tel Aviv, Israel), in which the aim was to imitate the movement of the wrist, elbow, and shoulder joint by whacking the ball with the racket into the glowing target as they appear; *Until You Fall* (Schell Games, Pittsburg, USA) game in which wrist flexion and extension with either pronation or supination of the forearm was to be executed for controlling and swinging the sword on an aesthetic battlefield; and *Holofit *game (Holodia, Strasbourg, France), where the patient concentrated on the rowing or skiing movement of the hand imitating the combination of flexion or extension with ulnar deviation or radial deviation. The upgrading of the level provides resistance to the interface.

The Oculus Quest-based rehabilitation or gamification was continued for four weeks, and a re-assessment was performed again after two weeks of gamification. The patient was also asked about her overall experience. It was reported that the patient felt comfortable using the head-mounted display and enjoyed serious gaming. Assessment after two weeks showed improvement in wrist ROM as shown in the readings reported at the end of the second week in Table 1. Grip strength improved from 4, which was on the first day, to 28 on the Jamar handheld dynamometer. The DASH score improved from 76.5 to 30 from pre-treatment to the two-week progression using gamification. 

After four weeks of gamification, patient progress was again recorded to determine the impact of gamification on pain, range of motion, grip strength, and hand functions. The results showed significant improvements in all previously recorded outcome measures. There was a convincing improvement in comparison with the first day of evaluation in wrist ROM to the readings recorded at the end of the fourth week as shown in Table 1. Grip strength improved from 4, which was on the first day, to 48 on the Jamar handheld dynamometer. The DASH score improved from 76.5 to 9.2** **from pre-treatment to the four-week progression using gamification.

## Discussion

Distal radius fracture presents with a complaint of pain and restricted ROM having a severe impact on the functional independence of the patient. The patient underwent rehabilitation using Oculus Quest HMD, which is a novel intervention providing an interface of an immersive virtual environment for rehabilitation [7]. The rehabilitation program involved three games namely the *Racket: Nx *game, *Until You Fall *game, and *Holofit *game imitating the normal movement of the hand and wrist across all three axes and planes. Gamification provides a virtual field that is an eminent expression in serious gaming [4, 8]. This provides an interesting, engaging, and motivating experience for the patient in exceeding their pain tolerance limits. The evolution of technology and the needs for painless procedures lay an open field for using gamification in rehabilitation to improve patient compliance and ultimately improve the outcomes of rehabilitation programs [9].

Serious games are being employed in non-traumatic conditions like non-significant chronic low back ache, in which a significant reduction in the intensity of pain and kinesiophobia had been observed [10]. Similarly, it has shown an eminent implication in neurological conditions like Parkinsonism by improving fine and gross coordination dexterity, strength, and speed of movement [11]. In traumatic brain injury, the application of virtual reality-induced gaming provides real-time capture of movements that can be freely moved in a simulated virtual environment [12]. It has shown significant improvement in shoulder stiffness by improving the kinetics and kinematics of shoulder joints using the real-time virtual feedback intervention [13]. Clinical implication of gamification in stroke has reported the benefits as easy access to upgrading difficulty level of tasks, standardized approach, and secure simulation with real-time feedback for upper limb motor rehabilitation [14]. In spinal cord injury patients, virtual reality-based rehabilitation is considered to have a positive impact on pain perception and motor function with a motivational imprint [15]. An immersive environment for the mobility of the wrist and hand induces progressive compliance and motivation for rehabilitation, making a significant impact visible in comparatively less duration. The engaging and interesting mode of gaming improves movement planning and its execution. Hence, gamification has an imperative effect on the rehabilitation of a patient recovering post-DRF.

## Conclusions

There was a substantial improvement with the implication of four weeks of gamification in pain, ROM, grip strength, and functional independence post-DRF. The use of the Oculus Quest modulated the rehabilitation program and enabled a broad array of therapeutic implications. The case report recommends access to exploring implications of serious gaming in the rehabilitation of other traumatic musculoskeletal conditions. The horizon of the implication of gamification can be explored by assessing the efficacy of gamification in randomized control trials on a larger sample size. Gamification can be used as an adjunct to conventional rehabilitation and improve the patient's compliance.
